# XY sex determination in a cnidarian

**DOI:** 10.1186/s12915-023-01532-2

**Published:** 2023-02-13

**Authors:** Ruoxu Chen, Steven M. Sanders, Zhiwei Ma, Justin Paschall, E. Sally Chang, Brooke M. Riscoe, Christine E. Schnitzler, Andreas D. Baxevanis, Matthew L. Nicotra

**Affiliations:** 1 School of Medicine, Tsinghua University, Beijing, China; 2 Visiting Scholar, School of Medicine, University of Pittsburgh, Pittsburgh, PA USA; 3 Starzl Transplantation Institute, Department of Surgery, University of Pittsburgh, Pittsburgh, PA USA; 4 Center for Evolutionary Biology and Medicine, University of Pittsburgh, Pittsburgh, PA USA; 5 Computational and Statistical Genomics Branch, National Human Genome Research Institute, National Institutes of Health, Bethesda, MD USA; 6 Whitney Laboratory for Marine Bioscience, University of Florida, St. Augustine, FL USA; 7 Department of Immunology, University of Pittsburgh, Pittsburgh, PA USA

**Keywords:** Pseudo-testcross, Pseudoautosomal region, *Hydractinia*, Sex determination, Linkage map, Depth of coverage

## Abstract

**Background:**

Sex determination occurs across animal species, but most of our knowledge about its mechanisms comes from only a handful of bilaterian taxa. This limits our ability to infer the evolutionary history of sex determination within animals.

**Results:**

In this study, we generated a linkage map of the genome of the colonial cnidarian *Hydractinia symbiolongicarpus* and used it to demonstrate that this species has an XX/XY sex determination system. We demonstrate that the X and Y chromosomes have pseudoautosomal and non-recombining regions. We then use the linkage map and a method based on the depth of sequencing coverage to identify genes encoded in the non-recombining region and show that many of them have male gonad-specific expression. In addition, we demonstrate that recombination rates are enhanced in the female genome and that the haploid chromosome number in *Hydractinia* is *n* = 15.

**Conclusions:**

These findings establish *Hydractinia* as a tractable non-bilaterian model system for the study of sex determination and the evolution of sex chromosomes.

**Supplementary Information:**

The online version contains supplementary material available at 10.1186/s12915-023-01532-2.

## Background

Sex determination in animals governs whether a gonad develops into an ovary or testis [1]. The primary sex determination signal can be genetic or environmental [2]. Genetic sex determination systems include sex chromosomes with male (XY) or female (ZW) heterogamety, haplodiploid systems in which males are haploid and females are diploid, or systems in which sex is determined by dosage at one or more autosomal loci. Environmental sex determination systems are similarly diverse, relying on external cues such as temperature, photoperiod, food, or social environment. In either case, the primary sex determination signal typically resides atop a cascade of pathways leading to the differentiation of either male or female gonads [3]. The fact that many genes from these pathways are conserved across animals has inspired hypotheses about how sex determination mechanisms evolve [4–6] and has also led to speculation about the nature of ancestral sex determination systems. This latter issue is difficult to address because most of what we know about animal sex determination comes from vertebrates, arthropods, nematodes, and a handful of other bilaterians [2, 7–9]. Therefore, it is important to investigate sex determination across a broader diversity of animal species, especially nonbilaterians.

Cnidarian sex determination systems would be particularly informative in this context, because the phylum is the sister group to all bilaterians [10, 11]. Cnidarians exhibit a range of sexual strategies, including gonochorism (separate sexes), simultaneous hermaphroditism, and sequential hermaphroditism [12]. Phylogenetic analyses indicate that the ancestral cnidarian was likely gonochoristic, with limited subsequent transitions to hermaphroditism throughout the phylum [13]. Although sexual differentiation and gametogenesis are well-studied in several cnidarian species [12], the primary sex determination signal has not been identified in any cnidarian. In fact, the only data directly addressing sex determination come from studies in two coral species. In the first, a karyotype of *Acropora solitaryensis* was characterized via fluorescence in situ hybridization with DNA extracted from sperm and eggs, revealing a putative Y chromosome [14]. In the second, a genome-wide analysis of single nucleotide polymorphisms (SNPs) from field-collected red corals (*Corallia rubrum*) yielded several male-specific loci consistent with chromosomal XX/XY sex determination [15].

**Fig. 1:**
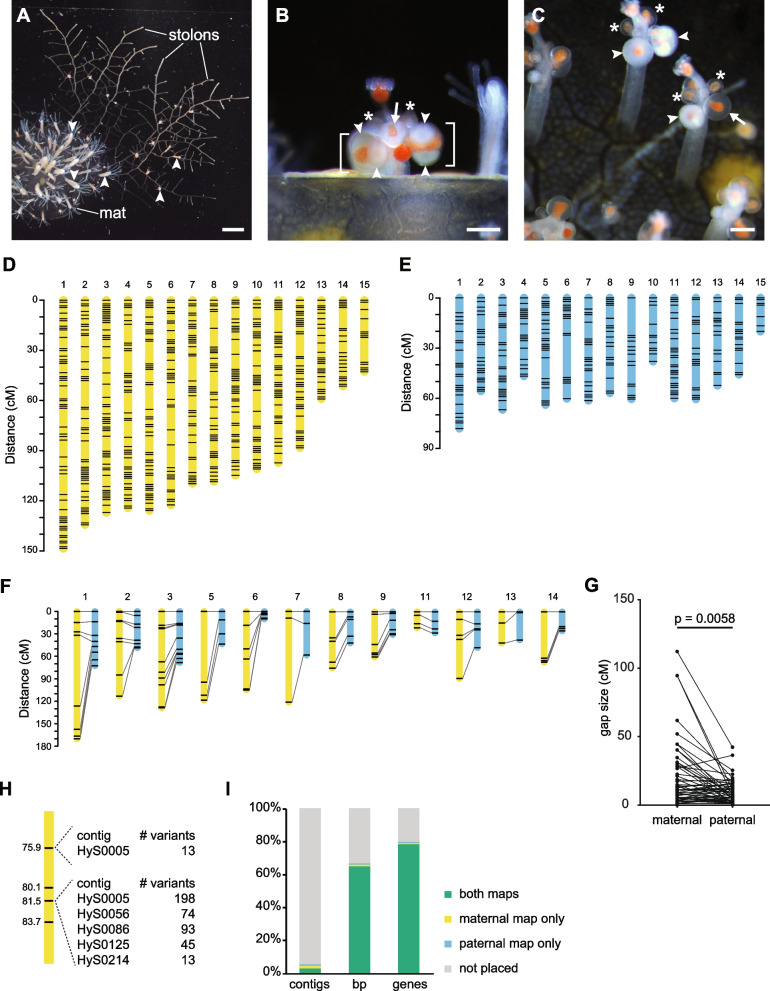
*Hydractinia* colonies and linkage maps. **A** An immature *Hydractinia* colony. Arrowheads indicate gastrozooids. Scale bar ≈ 1 mm. **B** Female gonozooid with mature gonophores (brackets) and eggs (arrowheads). Asterisks indicate immature gonophores. Scale bar = 200 μm. **C** Male gonozooids with mature (arrowheads) and immature (asterisks) gonozooids. Arrow indicates nearly mature gonophore. Scale bar = 200 μm. **D** Maternal linkage map. **E** Paternal linkage map. **F** Comparison of markers at equivalent physical locations in maternal and paternal maps. Lines connect markers located within 5 kb of each other in the reference genome. **G** Comparison of gap sizes in maternal and paternal linkage maps. Gap sizes from equivalent markers are connected by lines. **H** Example of how markers in the linkage map are bins of multiple variants. This example shows four markers from maternal linkage group 1. The marker at 75.9 cM represents 13 variants from one contig. The marker at 81.5 cM represents 423 variants from five contigs. **I** Representation of the *Hydractinia* genome assembly in the maternal and paternal linkage maps. Results are shown as a percent of the total number of contigs, base pairs (bp), or annotated genes

The hydroid *Hydractinia symbiolongicarpus* is a promising laboratory model system for cnidarian sex determination. *Hydractinia* colonies are gonochoristic and release gametes daily in response to a light cue. Fertilization is external, and each embryo develops into a crawling larva that metamorphoses into a primary feeding polyp. This polyp extends stolons from its base from which additional feeding polyps (gastrozooids) grow, thus creating a multi-polyp colony (Fig. 1A). Within 3–4 months, colonies develop polyps specialized for reproduction (gonozooids) in which eggs or sperm are easily observed (Fig. 1B, C). The presence of eggs or sperm is the only morphological difference between male and female gonozooids. In laboratory settings, experimental crosses produce offspring with a 1:1 sex ratio [16], but the primary sex determination signal is unknown. Most importantly, *Hydractinia* is a tractable genetic model system. It is easily bred in the laboratory, has a sequenced genome, and is amenable to gene knockdown, knockout, and knockin [17].

In *Hydractinia*, sexual differentiation and gametogenesis are processes that occur continuously throughout the colony’s life within each gonozooid [18, 19]. *Hydractinia* stem cells, known as i-cells, migrate into gonozooids, where they acquire germ cell fate and become gamete progenitors [20]. These progenitors then migrate from the neck of the gonozooid into sporosacs, where they mature into either eggs or sperm. Although it is unclear when sex determination takes place during this process, it is clear that the sexual identity of the colony is dependent on identity of the i-cell. Evidence for this comes from experiments in which a donor colony is grafted to an i-cell-depleted recipient that then begins to produce gametes matching the sex of the donor [19, 21, 22].

Although the sex of a colony is usually stable for its entire life, there have been reports of colonies that produce male and female gametes simultaneously [16, 23, 24]. In each case, the colony produces gonozooids with sporosacs containing mature sperm and immature eggs. In two cases, these colonies were reared from a single primary polyp, thus ruling out the possibility that male and female colonies had fused together and enabled i-cells of both sex to enter the same gonozooid [16, 24]. In our own laboratory, we have observed rare colonies that reach sexual maturity as fully functional females and then begin to produce male gametes that appear to overtake the entire colony (Leo Buss & Matthew Nicotra, unpublished observations). In these cases, it has been unclear whether the intersex colony is the product of an unnoticed fusion between males and females.

In this study, we sought to test the hypothesis that *Hydractinia* has genetic sex determination. To that end, we constructed a genome-wide linkage map and used it to identify sex-linked loci. We find a pattern consistent with XY sex determination. The Y chromosome is estimated to have half non-recombining regions and half pseudoautosomal regions. We then use a method based on sex-specific differences in whole genome resequencing depth of coverage to confirm the presence of an XY system and identify sex-linked loci. Of the 816 genes encoded in these sequences, we identify 70 that are exclusively expressed in the gonozooids of one sex, making them good candidates for *Hydractinia* sex determination genes.

## Results

### A linkage map of the *Hydractinia* genome

To create a *Hydractinia* linkage map, we generated a population of 90 F_1_ offspring by breeding a male colony (291–10) to a half-sibling female colony (295–8; Additional File 1: Fig. S1). Offspring and parents were then sequenced using Illumina sequencing methods and genotyped with the Genome Analysis Toolkit (GATK) [25]. After filtering the dataset to remove low-quality variants, we used a pseudo-testcross strategy [26] to generate separate maps of the maternal and paternal genomes.

The final map of the maternal genome consisted of 590 markers and spanned 1545.5 cM (Fig. 1D and Additional File 1: Fig. S2; Additional File 2), with an average gap of 2.69 cM/marker. The final map of the paternal genome consisted of 305 markers spanning 827.7 cM (Fig. 1E and Additional File 1: Fig. S3; Additional File 3), with an average gap of 2.85 cM/marker. Synteny between the maternal and paternal maps was determined by identifying linkage groups with a preponderance of markers from the same genomic contigs. Linkage groups were numbered according to decreasing genetic (cM) length in the maternal map. In both cases, 15 linkage groups were obtained.

To independently determine the number of chromosomes in *H. symbiolongicarpus,* we performed a karyotype analysis on metaphase cells isolated from embryos at the 64–128 cell stage. Nineteen out of twenty cells examined expressed a near-diploid chromosome complement with chromosome numbers of 30 per cell (2n = 30). One cell had a near-tetraploid chromosome number with an approximated 54 chromosomes. Another attempt to karyotype three cells revealed what appeared to be a normal diploid chromosome complement with 15 pairs of chromosomes (2n = 30) in the poorly G-banded metaphase cells (Additional file 1: Fig. S4). No dimorphic chromosomes were identified. Thus, karyotype and linkage analyses both predict a haploid chromosome number of 15 for *H. symbiolongicarpus.*

We immediately noticed that the maternal linkage map was nearly twice the length of the paternal map, suggesting the genome-wide recombination rate was higher in the female parent compared to the male. Alternatively, the maternal map might be longer because it incorporated more markers and was more likely to be inflated by genotyping errors [27]. To test these hypotheses, we compared rates of recombination between equivalent physical locations in the maternal and paternal genomes. To do this, we first identified pairs of markers — one maternal and one paternal — located within 5 kb of each other in the reference genome. We then used these markers to reconstruct each linkage group, allowing us to directly compare rates of recombination between nearly identical locations in the maternal and paternal genomes. Of the 12 reconstructed linkage groups, 11 were longer in the maternal map (Fig. 1F). Gaps in the maternal map were also larger than the equivalent gaps in the paternal map (*p* = 0.0058, Wilcoxon matched-pairs signed rank test; Fig. 1G) and the average gap size was larger in the maternal map (20.2 cM) than the paternal map (9.3 cM). These data were consistent with a higher recombination rate in the female genome across most chromosomes.

We next sought to determine how much of the *Hydractinia* genome assembly could be placed on each linkage map. Since each marker was a bin of variants from the reference genome, many markers represented variants from several contigs (Fig. 1H). To account for this, we created detailed linkage maps in which each marker was ‘unbinned’ and each underlying variant assigned a genetic position (Additional Files 4 and 5). We then determined how many contigs could be placed on each map. In all, 273 contigs, representing 65.2% of the 406,693,435 bp assembly and nearly 79.2% of the 22,022 annotated genes could be placed in at least one linkage map (Fig. 1I, Additional File 1: Table S1). This analysis also allowed us to identify 15 contigs that were split between different linkage groups and may represent misassemblies in the reference genome (Additional File 1: Table S2).

### *Hydractinia* has an XY sex determination system

**Fig. 2:**
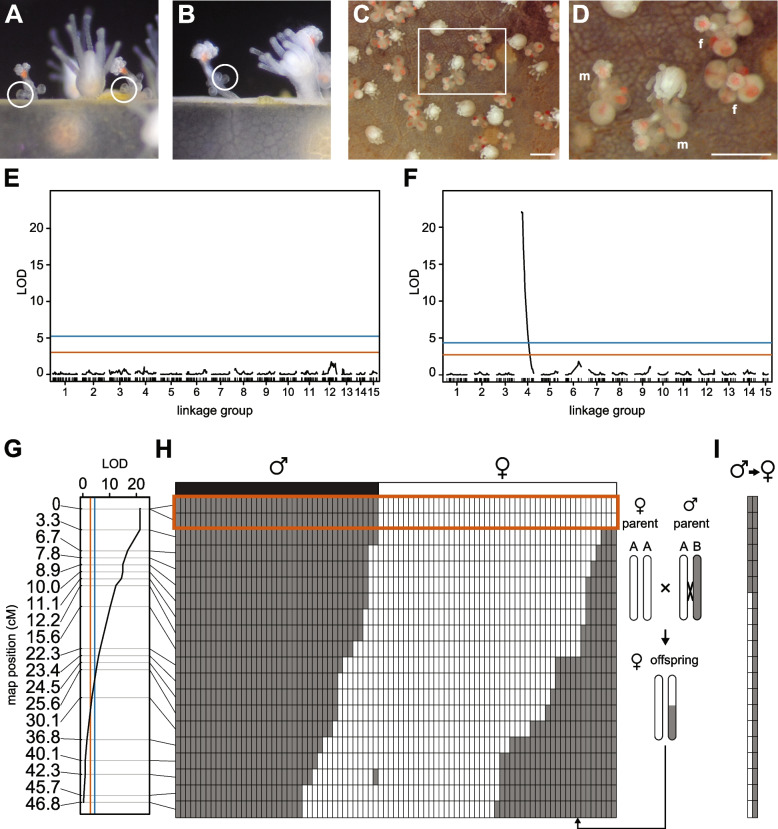
XY sex determination in *Hydractinia*. **A** Early and late stages of development of female gonozooids. **B** Early and late stages of development of male gonozooids. **C** Image of colony 339–116 on the day the first female gonophores were identified. **D** Close-up of boxed area in **C**. m, gonozooid bearing male gonophores. f, gonozooid bearing female gonophores. Scale bars in **C** and **D** are 1 mm. **E** LOD chart of QTL for sex in the maternal linkage map. **F** LOD chart of QTL for sex in the paternal linkage map. *(***G***)* Detail of LOD chart for linkage group 4 from the paternal linkage map. In **E**–**G**, significance thresholds of *p* = 0.05 and *p* = 10^−5^ are denoted in red and blue, respectively. **H** Recombination map of linkage group 4. Plot depicts genotype segregation pattern of pseudo-testcross markers (rows) in the F_1_ progeny (columns). Genotypes: AA: white; AB, gray. An example of a recombinant progeny is illustrated to the right of the plot. Observed sex of each F_1_ progeny is displayed above the plot. Genotypes in red box show perfect correlation with sex phenotype. **I** Recombination map of linkage group 4 in two animals with sexual chimerism

We recorded the sex of each F_1_ animal as soon as it could be determined from its developing gonophores (Fig. 2A–B). We then monitored the animals biweekly to identify instances of sexual chimerism. Most colonies (87/90) remained a single sex for the entire study, with an overall sex ratio that did not differ significantly from 1:1 (40:47 male:female; chi-squared goodness of fit test χ^2^ = 0.563, *p* = 0.453). In contrast, three male colonies appeared to change sex. Two began to develop female gonophores approximately six months after being classified as male (Fig. 2C and D). For both colonies, we explanted fragments bearing only male or female gonozooids to new slides and monitored them. These new colonies have remained either male or female for > 37 months. A third animal (339–083) was also initially classified as male but, six months later, was found to have only female gonozooids. While this animal may have undergone a complete sex change between our biweekly observations, we were unable to rule out the possibility that we had mislabeled it as male in our records, and this error had gone unnoticed for several months. We therefore excluded it from subsequent analyses.

We next used R/qtl [28] to identify markers associated with the initial sex of each animal. The analysis was performed independently on the maternal and paternal maps. While no significant loci were identified in the maternal map (Fig. 2E), several loci with a strong association with sex were located on the paternal map (Fig. 2F). The markers with the most significant association (*p* <  < 10^−5^) were located at 0 and 3 cM on linkage group 4 (Fig. 2G). Excluding the two sexual chimeras from this analysis did not affect these results significantly (Additional File 1: Fig. S5).

Next, we sought to determine how well the markers on linkage group 4 correlated with the initial sex of the colony. Using the linkage phase information estimated by OneMap, we determined the genotype of each F_1_ animal at each marker, which allowed us to infer which paternal haplotype had been inherited and whether it was a product of recombination (Fig. 2H). For animals with a stable sex, we found a perfect correlation between their sex and their genotype at the two 0 cM markers (Fig. 2H, red box). The two male-to-female chimeras also carried a “male” genotype at these markers (Fig. 2I). These data, combined with the fact that sex-linked markers were only identified on the male map, indicate *Hydractinia* has an XY sex determination system, and that the sex locus is located at the end of linkage group 4.

### The *Hydractinia* Y chromosome has a pseudoautosomal region

**Fig. 3:**
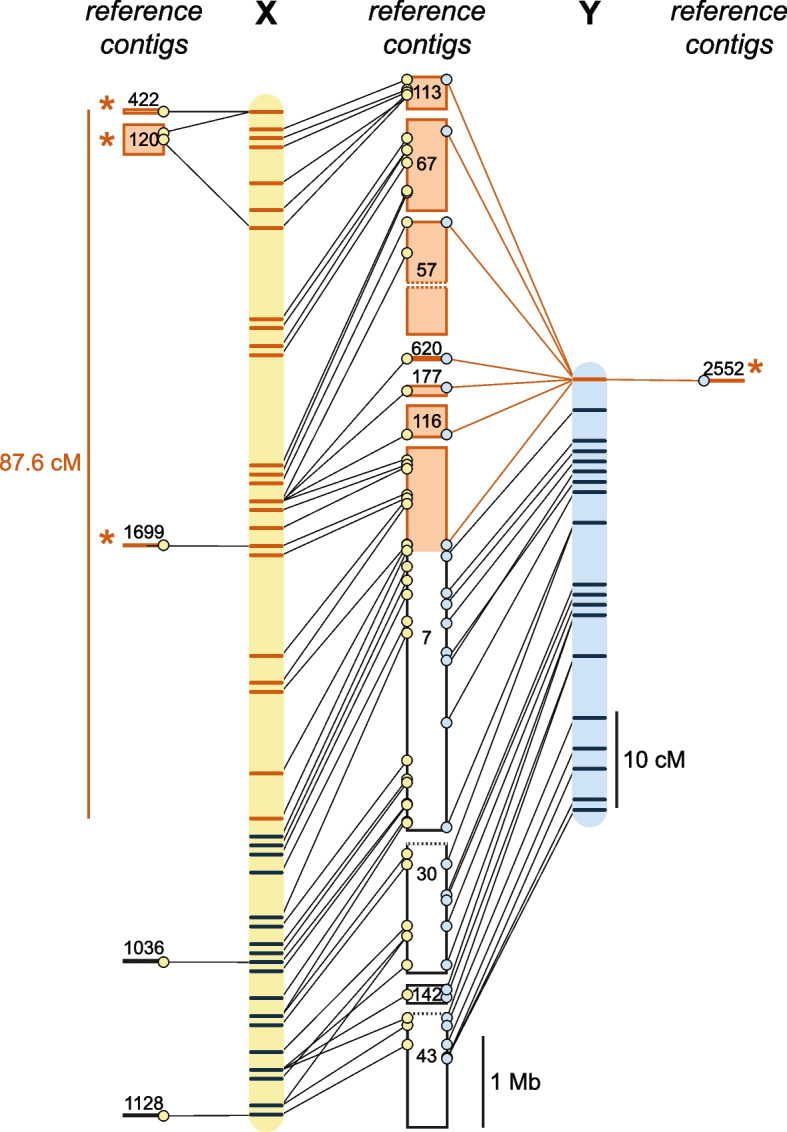
The *Hydractinia* X and Y linkage groups and sequences. Genomic positions of markers in the maternal (yellow) and paternal (blue) maps of linkage group 4. Regions and markers outlined in orange are located within the non-recombining regions of the X and Y chromosome. Markers indicated in black are in the pseudoautosomal region. Dotted lines indicate contigs 30 and 43 are each probably misassembled from separate linkage groups (see Supplemental Table 2). Asterisks indicate contigs in the non-recombining region that were only placed in one linkage map

As sex chromosomes evolve from autosomes, the sex-limited chromosome (Y or W) typically develops recombination suppression followed by degeneration and gene loss in the non-recombining region [29]. This non-recombining region is often linked to a pseudoautosomal region that continues to recombine with its heterologous counterpart (X or Z). To determine the extent of these regions on the *Hydractinia* Y chromosome, we drew connections between the maps of linkage group 4 and the reference genome assembly (Fig. 3). To simplify the visualization, each marker was mapped to the physical position of one representative variant per contig. Six contigs from the reference assembly could only be connected to one of the linkage maps. These contigs may represent sequences that have significantly diverged or been lost on either the X or the Y chromosome. Alternatively, they may not have been placed in both maps because they did not possess heterozygous variants in one parent.

On the paternal map, which represents recombination between the X and Y chromosomes, the two 0 cM markers corresponded to a non-recombining region spanning at least 6.46 Mbp. This same genomic region corresponded to 87.6 cM on the maternal linkage map, indicating ample recombination between X chromosomes in the maternal genome. The pseudoautosomal region spanned at least 10.6 Mbp and corresponded to 36.7 cM in the maternal map and 46.8 cM in the paternal map. Together, these data indicate that more than half of the *Hydractinia* Y chromosome is a pseudoautosomal region.

### Depth of coverage method confirms XY sex determination and identifies additional sex-linked sequences

We suspected some of the unmapped contigs in the genome might be from sex chromosomes. One way to identify such sex-linked sequences in whole genome sequencing datasets is to take advantage of differences in the depth of coverage (DoC) between males and females [30]. In an XY sex determination system, sequences on the X chromosome are expected to have twice the DoC in female samples compared to male samples.

Recently, a method called “sex assignment through depth of coverage” (SATC) has been developed can identify sex-linked sequences using only a reference assembly and whole genome sequencing data from a population that includes both sexes [31]. The method normalizes the DoC of each contig across all samples such that the value for most contigs is 1.0. It then uses a principle component analysis of the variation in DoC to classify each sample as either heterogametic or homogametic. Finally, it uses this classification to identify contigs with statistically significant differences in their mean DoC between the two sexes. Contigs with a mean difference close to 0.5 (between 0.4 and 0.6) are flagged as highly likely to be on the X chromosome. Other contigs with significant differences are flagged as “sex-linked or abnormal”. Some of these may be X-linked, while others may be Y-linked and therefore have little to no coverage in female samples.

**Fig. 4:**
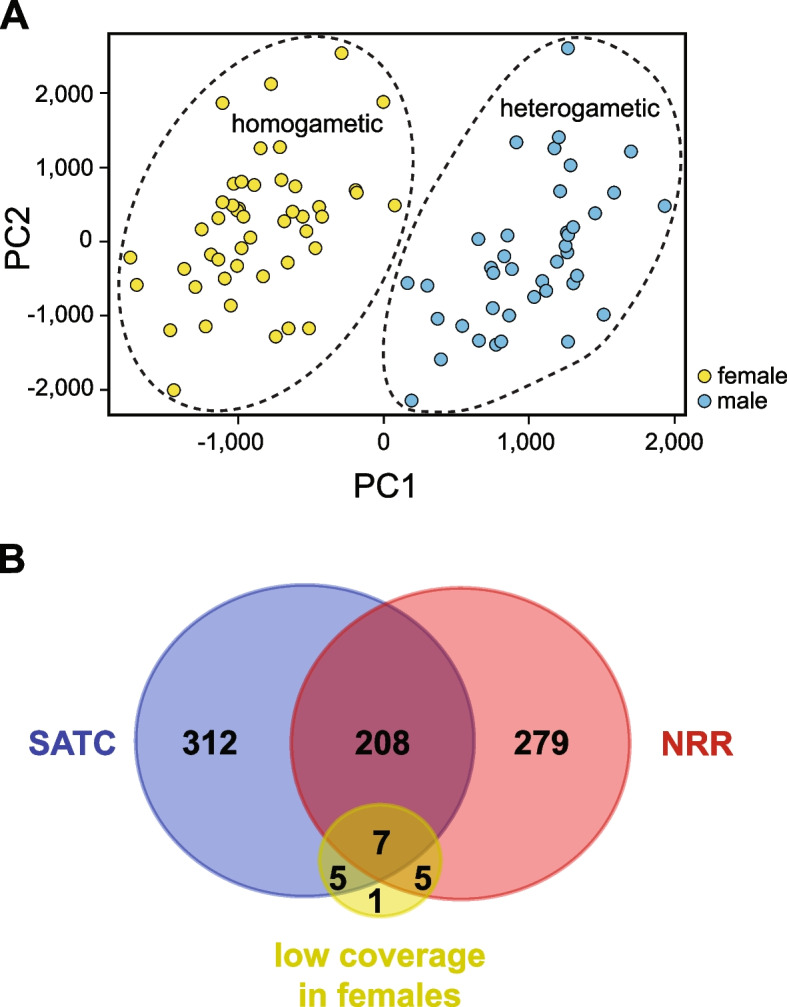
SATC analysis. **A** Principal component analysis of variance in depth of coverage between samples. Groups predicted to be homogametic or heterogametic by SATC are indicated with dashed lines. Yellow circles are samples phenotyped as females, blue circles are samples phenotyped as males. **B** Venn diagram of the number of genes located in the non-recombining region of linkage group 4 (NRR), on SATC-flagged contigs (SATC), or having an unusually low depth of coverage in females. (Diagram created with https://bioinformatics.psb.ugent.be/webtools/Venn/)

Because SATC was specifically developed for fragmented genomes of non-model organisms [31], we reasoned we could use it to identify additional sex-linked contigs in the *Hydractinia* genome. We found that SATC correctly determined the sex of all samples in our mapping population (Fig. 4A). This independently confirmed our conclusion that *Hydractinia* has an XY sex determination system and increased our confidence that the method would correctly identify unmapped contigs with sequences from sex chromosomes.

Indeed, SATC identified 137 contigs with statistically significant differences in DoC between males and females (Additional File 6: Table S3). Ten contigs were in linkage group 4. Three were in other linkage groups and were excluded from further analysis. Of the remaining 124 contigs, 108 were flagged as “sex-linked or abnormal”, and 16 were flagged as X-linked. In total, these 124 new contigs represented 6,908,237 bp of additional sequence putatively from the *Hydractinia* sex chromosomes*.*

Y-linked genes are expected to have little to no coverage in WGS datasets from female samples. We therefore searched for genes with a significant deficit in coverage in the female samples. These may not have been flagged by SATC if they were on contigs that were chimeras of X- and Y-linked genes. To account for this, we created a separate DNA sequence file for each genomic locus and mapping the WGS data to each one individually. We then searched for genes with < 50% coverage in one sex and > 90% coverage in the other. This revealed 18 genes with low coverage in females compared to males (Additional File 1: Table S5). Twelve genes were located on contigs HyS0007, HyS0057, HyS0067, and HyS00113, which are part of the non-recombining region of the linkage map (Fig. 3 and Additional File 8). Five of the remaining were from contig HyS0070, which was flagged as sex-linked by SATC and is therefore likely to be in the non-recombining region as well. The last gene, HyS3947.3 was not identified in any other analysis (Additional File 8: Table S4). We did not identify any genes with > 90% coverage in females and < 50% coverage in males. These results are consistent with XY sex determination in *Hydractinia.*

### Gene content of the *Hydractinia* sex locus

Our data indicate that the gene(s) controlling sex determination are located within the non-recombining region of the X and Y chromosomes. This region includes the 499 genes encoded on contigs in the non-recombining region of the linkage map plus an additional 317 from the putative sex-linked contigs identified by SATC, and one additional gene lacking coverage in females (Fig. 4B; Additional File 7). For the sake of completeness, we include all 817 genes even though some may not be in the non-recombining region. Annotation of these genes revealed a variety of predicted functions (Additional File 8: Table S4) but no obvious homologs of genes or domains found in other animal sex determination pathways.

A reasonable assumption for a sex determination gene is that it is only expressed in the gonozooids of one sex. Therefore, we used previously published data [32–35] to calculate the average expression of each gene in gastrozooids, male gonozooids, and female gonozooids. When we set a threshold of > 1 fragment per kilobase per million mapped reads (FPKM) for a gene to be considered “expressed,” we identified nine genes expressed only in female gonozooids and 29 genes expressed only in male gonozooids (Table 1 and Additional File 8: Table S4). The remaining genes were either expressed in both sexual polyp types, expressed in gastrozooids, or unexpressed (Additional File 1: Table S6 and Additional File 8: Table S4). During our analysis, we noticed several genes with high expression in either male or female gonozooids but very low expression in gastrozooids. Therefore, we ran a second calculation using 5 FPKM as the expression threshold. At this level, we identified an additional 9 female gonozooid-specific and 23 male gonozooid-specific genes (Table 2). In both analyses, there were more male gonozooid-specific than female gonozooid-specific genes and, moreover, the male-specific genes were more highly expressed than the female-specific ones.

**Table 1:** Candidate sex determination genes with gonozooid- and sex-specific expression at an expression threshold of 1 FPKM

	Expression (FKPM)			Analysis			
	*Gastro*	*Female gono*	*Male gono*	*NRR*^a^	SATC^b^	*Female coverage deficit*^c^	*Comment*^d^
HyS1036.2	0.15	**22.62**	0.47		x		Helix-loop-helix domain-containing protein
HyS2624.2	0.00	**12.47**	0.00		x		*Unknown function*
HyS0007.332	0.00	**9.66**	0.00	x			*Unknown function*
HyS0116.3	0.00	**5.77**	0.00	x	x		*Unknown function*
HyS0070.121	0.00	**2.19**	0.30		x		Reverse transcriptase-like protein
HyS0120.5	0.00	**1.64**	0.00	x	x		*Unknown function*
HyS0328.2	0.00	**1.05**	0.00		x		*Unknown function*
HyS0070.32	0.51	**1.03**	0.14		x		Headcase protein
HyS0007.350	0.36	**1.35**	0.87	x			*Unknown function*
HyS0070.46	0.34	0.11	**358.41**		x		*Unknown function*
HyS0057.51	0.84	0.03	**314.84**	x			WH1/EVH1 domain-containing protein
HyS0057.46	1.00	0.00	**192.60**	x			Cyclic nucleotide-binding domain-containing protein
HyS0007.291	0.27	0.15	**115.53**	x			*Unknown function*
HyS0057.47	0.00	0.00	**67.81**	x			Potassium/sodium hyperpolarization-activated cyclic nucleotide-gated channel
HyS0067.10	0.16	0.00	**30.26**	x	x		*Unknown function*
HyS0070.23	0.45	0.00	**27.85**		x		*Unknown function*
HyS0070.81	0.07	0.00	**18.79**		x		*Unknown function*
HyS0113.16	0.76	0.00	**6.41**	x	x	x	*Unknown function*
HyS0067.3	0.45	0.03	**5.67**	x	x		Likely KDZ transposase
HyS0113.15	0.00	0.00	**4.61**	x	x	x	*Unknown function*
HyS0067.73	0.00	0.00	**4.37**	x	x		*Unknown function*
HyS0069.15	0.50	0.52	**4.75**		x		ATP-dependent helicase-like
HyS0007.328	0.27	0.24	**3.76**	x			Cupin-domain-containing protein
HyS0057.18	0.00	0.00	**3.30**	x			*Unknown function*
HyS0067.71	0.59	0.00	**2.85**	x	x		*Unknown function*
HyS4443.1	0.37	0.16	**2.20**		x		*Unknown function*
HyS0057.124	0.00	0.09	**1.81**	x		x	COUP-TF-like nuclear hormone receptor
HyS0057.129	0.31	0.12	**1.82**	x			COUP-TF-like nuclear hormone receptor
HyS0069.70	0.00	0.00	**1.64**		x		*Unknown function*
HyS1036.1	0.03	0.31	**1.82**		x		U3 small nucleolar ribonucleoprotein complex, subunit MPP10
HyS0070.5	0.14	0.00	**1.45**		x		Ankyrin repeat-containing protein
HyS0116.7	0.93	0.91	**2.20**	x	x		HECT domain-containing protein
HyS0069.45	0.00	0.00	**1.19**		x		*Unknown function*
HyS0057.71	0.67	0.06	**1.20**	x			CXC domain-containing protein-like
HyS0067.4	0.06	0.00	**1.07**	x	x		Brinker DNA-binding domain containing protein
HyS0007.327	0.00	0.00	**1.02**	x			*Unknown function*
HyS0070.97	0.97	0.17	**1.07**		x		*Unknown function*
HyS0057.113	0.44	0.51	**1.27**	x	x		Similar to SCAN-domain-containing protein

^a^Is in the non-recombining region of linkage group 4

^b^Flagged as sex-linked by SATC

^c^Gene with a deficit in coverage in females vs males

^d^Authors’ comment on potential homology or function of gene product

**Table 2:** Candidate sex determination genes with gonozooid- and sex-specific expression at an expression threshold of 5 FPKM

	Expression (FKPM)			Analysis			
	*Gastro*	*Female gono*	*Male gono*	*NRR*^a^	*SATC*^b^	*Female coverage deficit*^c^	*Comment*^d^
HyS2624.1	2.43	**211.27**	1.89	-	x		*Unknown function*
HyS0007.282	0.87	**31.75**	1.14	x			*Unknown function*
HyS0069.11	2.60	**26.69**	1.83	-	x		*Unknown function*
HyS0918.1	4.10	**15.36**	4.17	-	x		Kinase-BAR domain-SH3-coiled coil domain-containing protein
HyS0148.2	3.15	**10.27**	2.70	-	x		*Unknown function*
HyS4472.1	1.91	**5.44**	0.04	-	x		Gamma-glutamyltranspeptidase
HyS0067.50	1.91	**5.21**	0.58	x	x		FGF-receptor/Basigin-like protein
HyS0067.52	2.85	**5.66**	1.03	x	x		FGF-receptor/Basigin-like protein
HyS0007.322	3.09	**8.03**	4.23	x	-		Importin-11 like
HyS0007.412	2.83	0.40	**943.95**	x	-		*Unknown function*
HyS0057.61	2.08	0.00	**859.64**	x	-		E3 ubiquitin-protein ligase MARCHF2/3-like
HyS0057.48	2.84	0.04	**531.51**	x	-		Potassium/sodium hyperpolarization-activated cyclic nucleotide-gated channel
HyS0057.36	4.26	0.27	**475.83**	x	-		*Unknown function*
HyS0070.65	1.93	0.05	**376.95**	-	x		DBF4 zinc finger-like protein
HyS0007.262	2.50	3.75	**194.30**	x	-		Testis-Expressed Protein 45-like
HyS0057.16	3.91	3.05	**32.44**	x	-		Protein FAM221B-like
HyS0070.25	3.27	1.73	**31.05**	-	x		*Unknown function*
HyS0113.24	4.63	0.06	**12.41**	x	x		Pancreatic trypsin inhibitor Kunitz domain-containing protein
HyS0057.114	2.15	0.21	**11.43**	x	-		Zinc-finger domain-containing protein
HyS0007.257	1.64	0.98	**11.43**	x	-		Glycosyl transferase, family 31
HyS0120.42	2.88	0.35	**9.24**	x	x		SET domain-containing protein
HyS0007.368	3.36	0.24	**8.58**	x	-		Thrombospondin repeat domain-containing protein
HyS0067.49	0.80	2.18	**10.50**	x	x		Ankyrin repeat and LEM domain-containing protein
HyS0070.83	3.02	0.23	**8.50**	-	x		Protein patched/dispatched family member
HyS0067.65	1.39	0.27	**7.65**	x	x		Alpha-2 macroglobulin-like
HyS0007.261	1.67	0.20	**7.50**	x	-		Cytochrome P450, E-class, group I
HyS0057.88	2.81	0.19	**6.35**	x	-	x	Ubiquitin domain-containing protein
HyS0070.60	4.87	2.62	**7.85**	-	x		Phosphatidylinositide phosphatase SAC2
HyS0244.4	3.75	1.98	**6.79**	-	x		Elongation factor-like GTPase 1
HyS1699.2	4.16	2.40	**5.84**	x	x		Fanconi-associated nuclease 1-like
HyS0007.387	2.71	3.01	**5.09**	x	-		DNA topoisomerase 3-like
HyS0070.70	4.29	3.84	**5.76**	-	x		F-box and LRR domain containing protein

^a^Is in the non-recombining region of linkage group 4

^b^Flagged as sex-linked by SATC

^c^Gene with a deficit in coverage in females vs males

^d^Authors’ comment on potential homology or function of gene product

## Discussion

In this study, we demonstrate that *H. symbiolongicarpus* has an XX/XY genetic sex determination system via two orthogonal methods: linkage mapping and depth of coverage analysis. Our linkage map shows the Y chromosome has sizeable pseudoautosomal and non-recombining regions. The non-recombining region spans at least 6.46 Mbp and contains 499 genes. An additional 6.91 Mbp, encoding 317 genes, are highly likely to be sex-linked according to DoC analysis, although some may be located outside of the non-recombining region. Of these 816 genes, 70 are expressed exclusively in either male or female gonozooids, making them good candidates for *Hydractinia* sex determination genes.

Although *Hydractinia* has sex chromosomes, how they function to determine sex remains unclear. Sex determination via X and Y chromosomes can occur in several ways. In mammals, for instance, the Y chromosome encodes the *sex determining region Y* (*Sry*) gene, a transcription factor that is the only gene required to initiate the formation of testis in bipotential gonad [36]. On the other hand, in *Drosophila* the Y chromosome plays no role in sex determination. Rather, it is the number of X chromosomes that determines whether a cell is male or female [37]. Cells “count” the number of X chromosomes via the concentration of four X-linked proteins that, above a certain threshold, allow the “master switch,” *Sex-lethal*, to be activated. Further study will be required to determine whether *Hydractinia* follows one of these strategies or an alternative. Until then, we think it is reasonable to consider genes with sex-specific gonozooid expression to be good candidates.

Another question is when and where sex determination occurs in *Hydractinia*. *Hydractinia* colonies continue to grow new gonozooids throughout their lives. Within each, i-cells continuously differentiate into germline progenitors [18–20]. One possibility is that the sex determination pathway is activated each time an i-cell commits to gametogenesis. This would likely occur within the germinal zone of the gonozooid after i-cells begin to express *Tfap2*, a transcription factor that commits i-cells to a germ cell fate [20].

Regardless of the mechanism and location of sex determination, our data indicate the sex determining factor(s) lie within the non-recombining region of the X and Y chromosomes. We estimate this region contains 499 genes, plus at least a portion of those identified as sex-linked by SATC. Thus, a crucial question is whether SATC has correctly identified all sex-linked genes in the genome. We suspect it has not because it failed to identify two contigs in the non-recombining region (HyS0007 and HyS0057) as sex-linked. It also misidentified three autosomal contigs as “sex-linked or abnormal”. Our list of 816 genes probably includes false positives and is missing some sex-linked genes. Moreover, we cannot be certain which genes are X-linked vs Y-linked because the reference genome was assembled from a male colony. Thus, each contig in linkage group 4 could represent an X-specific DNA sequence, a Y-specific DNA sequence, the consensus sequence of a region conserved between X and Y, or a patchwork of X- and Y-specific regions connected by conserved sequences. With these caveats in mind, our analysis of the *Hydractinia* sex locus should be viewed as a “first look” to inspire future studies [34].

One takeaway from this first look is that the sex locus does not appear to contain any of the so-called ‘usual suspects’ for metazoan sex determination genes [6]. These include Sox family transcription factors and parts of the TGF-beta signaling pathway in vertebrates, *transformer* genes in insects, and *feminizer* genes in a variety of invertebrates. It also includes “Doublesex and Mab-3” (DM) domain-containing genes, which have been found in the sex differentiation pathways of most species studied to date [6, 38]. Narrowing our focus to genes expressed only in the gonozooids of one sex identifies 70 genes which, in our opinion, are top candidates for a sex-determination locus. Several of these are proteins with sequence similarity to transcription factors and other DNA binding domains and may therefore be attractive targets for future studies. One in particular, a COUP-TF-like hormone receptor, which appears to be absent in females, may deserve special attention.

The lack of obvious candidates raises the question of whether the *Hydractinia* sex determination pathway is homologous to the pathways in other animals. Thus, it is germane to note that DM domain-containing genes have been identified in most animal genomes, including cnidarians [39]. In addition, at least one DM domain-containing gene has male gonozooid-specific expression, although its function has not been determined [20]. Thus, *Hydractinia* might follow a familiar evolutionary pattern where the overall sex determination and differentiation pathways are conserved and a gene from that pathway has been coopted as the primary sex determination signal [40]. Alternatively, the primary sex determination signal could be a novel one. This would not be unprecedented; in salmonids, the master sex determining gene *sdY* evolved via duplication from *interferon regulatory factor 9*, an immunity-related gene with no known function in gonad development [41, 42].

Another question is how the X and Y chromosome suppress recombination. Several mechanisms for recombination suppression have been proposed, including sexual antagonism [43, 44], meiotic drive [45], and heterozygote advantage [46]. Further study of the sex chromosomes in *Hydractinia* and related cnidarians may reveal some answers. One consequence of recombination suppression is the accumulation of repetitive DNA in the non-recombining regions of the sex-limited (Y) chromosome. We find a modest increase in repetitive DNA in the non-recombining region of the *Hydractinia* Y chromosome compared to the pseudo-autosomal region and the autosomes. This finding will need to be validated once chromosome-level assemblies of the *Hydractinia* genome are produced such that all repeats can be assigned to linkage groups.

The observation of non-recombining and pseudoautosomal regions in the X and Y chromosomes raises the possibility that they may be dimorphic. Although our karyotype analysis revealed 15 pairs of chromosomes (2n = 30), the G-banding was poor, and we did not observe any obviously dimorphic pairs. Future experiments using FISH to mark sex chromosomes with sex-specific probes will be necessary to identify and then characterize the morphology of the X and Y chromosomes.

One perplexing finding was the presence of two colonies that were initially classified as male but later observed with male and female gonozooids. Male- and female-specific explants from these chimeras have remained exclusively one sex for more than 3 years. The direction of this sexual chimerism (male to female) is different from that which has been reported previously [16, 23, 24]. Together, these observations suggest that, although *Hydractinia* has genetic sex determination, aberrations may occur that cause gonads to produce both eggs and sperm or colonies to produce both male and female gonozooids. One possible mechanism for this could be mutations in a subpopulation of i-cells that alters their sexual identity. An alternative explanation in the case of our study is that each sexual chimera was the product of an undetected fusion between juvenile male and female colonies, where i-cells from both sexual identities persisted in the chimera, but the male matured sooner than the female.

## Conclusion

In summary, we have used linkage mapping and a depth of coverage approach to demonstrate that *Hydractinia* has XY sex determination. We then identified ~ 13.3 Mb of sequence encoding candidates for the primary sex determination signal, including 70 with sex-specific expression in gonozooids. This work, which maps a sex locus in a genetically tractable cnidarian is a major step toward elucidating sex determination pathways in a broader diversity of animals. With these tools in hand, interested scientists should be able to generate data to bring to bear on questions regarding the sex determination outside of bilaterians in during early animal evolution.

## Methods

### Breeding and animal maintenance

Colonies were maintained at the University of Pittsburgh as described in [47]. Briefly, they were grown on 75 mm × 25 mm glass slides in 38-l aquaria filled with artificial seawater (Instant Ocean Reef Crystals) and maintained at 22–23 °C. Adult colonies were either fed 3-day-old Artemia nauplii (3 × per week) or a suspension of pureed oysters (× per week). Breeding colonies were kept on a 8-h/16-h light/dark cycle. After their first exposure to light, males and females were placed in separate 3-l bins, where they released gametes 1–1.5 h later. Within 20 min of spawning, eggs were harvested by filtering water from the female bin through a 20-μm cell strainer, and sperm was harvested by collecting 10–15 ml from the male container. Eggs were fertilized by mixing them with the sperm and 15 ml of additional artificial seawater in a 100-mm polystyrene petri dish. Embryos developed into larvae and were settled 72–96 h post-fertilization (hpf). Metamorphosis was induced by incubating the larvae in 56 mM CsCl in filtered seawater for 4–6 h, then transferring them with glass pipettes onto glass microscope slides. Three days later, larvae that successfully metamorphosed began feeding as described above, but without the oyster supplement.

### Mapping population and sample preparation

A mapping population was created by crossing a male colony (291–10) to a female half-sibling (295–8). The resulting F_1_ offspring were observed weekly until male or female gonophores could be discerned, at which point they were scored appropriately and moved to male-only or female-only tanks. Thereafter, colonies were observed and cleaned biweekly.

DNA was extracted from colonies when they had grown to cover approximately 2 cm^2^ of their slide. In most cases, this was performed before the animals had been classified as male or female. Animals were starved for at least two days, then a portion of the colony measuring ~ 1 cm^2^ was removed by scraping it from the slide with a razor blade. Harvested tissue was placed in a 1.7-mL microfuge tube, briefly spun in a benchtop microcentrifuge, and residual seawater removed by aspirating with a pipette. Tissue was lysed by the addition of 200 μL UEB buffer (7 M Urea, 0.3125 M NaCl, 0.05 M Tris–HCl pH 8.0, 0.02 M EDTA pH 8.0, 1% N-Lauroylsarcosine sodium salt) [48], followed by grinding with a plastic pestle until all tissue was dissolved. Next, one volume of equilibrated phenol:chloroform:isoamyl alcohol (25:24:1) was added and the mixture homogenized by inverting vigorously. The mixture was centrifuged for 10 min at > 3000 g, and the aqueous phase transferred to a new tube. Total nucleic acid was precipitated with 0.7 volumes of isopropanol, then centrifuged for 30 min at top speed (at least 13,000 rpm) at room temperature. The resulting DNA pellet was washed twice with 70% ethanol, then transferred to a new tube, centrifuged for 1 min, and excess 70% ethanol aspirated with a pipette. To remove RNA, the DNA pellet was resuspended in 1X TE, and 1 μL Ambion Rnase cocktail (ThermoFisher, Cat. AM2286) per 100 μL suspension was added, followed by incubation at 37 ℃ for 15 min. DNA was then re-extracted with phenol:chloroform:isoamyl alcohol (25:24:1), precipitated with isopropyl alcohol, washed with ethanol, and resuspended with 1XTE as described above. DNA samples were stored at − 20 °C prior to being sent to the NIH Intramural Sequencing Center for sequencing.

### Karyotype analysis

Embryos from a cross between colony 291–10 and colony 295–8 were used to generate multiple embryos for karyotyping. Embryos at the 64–128 cell stage (approximately 8 h post-fertilization) were submitted to the University of Pittsburgh Cell Culture and Cytogenetics Facility. The embryos were split into three tubes with 3 ml of seawater each. Two tubes were treated with 60 µl ColcemidTM (0.1 µg/mL) and one tube was treated with 60 µl vinblastine (0.01 mg/ml) (f.c. 0.2 ng/µl) for 90 min with gentle shaking. Following mitotic arrest, one Colcemid tube was treated with 5 ml 1:1 (sterile water:Mg + Ca + free seawater) hypotonic solution with 50 µl Colcemid for 30 min with constant agitation; the vinblastine tube was treated with 5 ml 1:1 (sterile water:Mg + Ca + -free seawater) hypotonic solution with 50 µl vinblastine for 30 min with constant agitation, and the third tube with 90 min Colcemid was treated with 0.075 M KCl with 50 µl Colcemid for 30 min. Tubes were fixed with Carnoy’s fixative and stored at − 20 °C overnight. Slides were prepared the next day following a few washes in the fixative. Both Colcemid-treated samples produced a few metaphase cells, but the vinblastine tube produced no visible metaphase cells. Slides were observed on an Olympus BX61 microscope and imaged and analyzed using the Genus software platform on the Cytovision System (Leica Microsystems, San Jose, CA).

### Whole genome sequencing and SNP discovery

A detailed description of the complete analysis pipeline, including all scripts, can be downloaded from https://github.com/nicotralab/chen-et-al-sex-determination [49]. Several data sets were too large to be placed in the GitHub repository but can be downloaded directly from https://zenodo.org/record/6368105 [50]. The reference assembly of the *Hydractinia* genome used in this study was that of the male parent, colony 291–10 [51, 52]. It was assembled from a combination of PacBio long-read and Illumina-short read sequencing and consisted of 4,840 scaffolds, with an N50 of 2.2 Mb. Raw reads are available via BioProject PRJNA807936. The version of the assembly used in this project can be downloaded from https://zenodo.org/record/6368105 [50].

To generate sequence data for variant calling for the female parent and all F_1_ progeny, PCR-free libraries were generated from 1 µg genomic DNA using the TruSeq® DNA PCR-Free HT Sample Preparation Kit (Illumina). The median insert sizes were approximately 400 bp. Libraries were tagged with unique dual index DNA barcodes to allow pooling of libraries and minimize the impact of barcode hopping. Libraries were pooled for sequencing on the NovaSeq 6000 (Illumina) to obtain at least 500 million 151-base read pairs per individual library. We obtained a mean coverage of 47 ± 11 × per sample, with a mean depth of 162 ± 38 million reads per sample (for per-sample statistics, see the “Methods” section and Additional File 9).

All raw sequence data for the female parent and offspring are available via BioProject PRJNA816479 at NCBI. For the male parent, we used Illumina sequencing data from the genome project. The original Illumina dataset, which consisted of 240 million reads, was downsampled with seqtk [53], resulting in 66 × 10^6^ 251 bp paired end reads. The downsampled files can be downloaded from https://zenodo.org/record/6368105 [50].

For each sample, raw reads were mapped to an assembly of the paternal genome. Reads were mapped to the assembly using BWA-MEM [54] with mapping parameters “-M -t 8.” The resulting.sam files were converted to.bam format and then sorted with Samtools [55]. Duplicates were then marked with Picard [56]. Genotypes were called with GATK HaplotypeCaller [25]. The resulting file, rawvariants.90f1.vcf.gz, can be downloaded from https://zenodo.org/record/6368105 [50]. A total of 9.74 million single nucleotide polymorphisms (SNPs) and 1.83 million insertion/deletion variants (indels) were identified.

Raw variant calls were filtered to generate datasets of high-quality variants suitable for genetic mapping. Briefly, raw variant calls were filtered with a custom python script (qualityfilter.py) to retain only those variants for which (1) no samples were missing data; (2) all samples genotyped as homozygous reference (0/0) had no more than two mapped reads corresponding to the alternative allele and also had more than ten mapped reads corresponding to the reference; (3) all samples genotyped as homozygous alternative (1/1) had no more than two mapped reads corresponding to the reference allele and also had more than ten mapped reads corresponding to the alternate allele; and (4) all samples genotyped as heterozygous (0/1) had an alternate allele read count percentage of greater than 0.3 or less than 0.7. This dataset was further filtered according to GATK best practices [57]. Specifically, SNPs were flagged as low quality if they met any of the following criteria: quality by depth (QD) < 2; Fisher’s exact test of strand bias (FS) > 60; RMS mapping quality (MQ) < 40; rank sum of alt versus reference mapping quality (MQRankSum) < 12.5; read position rank sum (ReadPosRankSum) < 8; and read depth (DP) < 10. Indels were flagged as low quality if they met any of the following criteria: QD < 2.0, FS > 200, or ReadPosRankSum <  − 20.0. Flagged variants were then removed with bcftools [55]. The resulting file, GATK-passed.vcf, can be downloaded from https://zenodo.org/record/6368105 [50]. After this filtering, 1,312,632 variants (1,083,937 SNPs and 228,695 indels) remained.

From this filtered dataset, we generated two sets of markers suitable for mapping via a pseudo-testcross strategy [26]. In a pseudo-testcross, two parents from an outcrossing population are bred to create an F_1_ population. A genetic map for each parental genome is then constructed with markers that are heterozygous in that parent and homozygous in the other. For example, a genetic map of the maternal genome can be constructed from variants that are homozygous in the male parent and heterozygous in the female parent (i.e., “0/0” × “0/1” in the notation of a.vcf file). Likewise, a genetic map of the paternal genome can be constructed from variants heterozygous in the male parent and homozygous in the female parent (e.g., “0/1” × “0/0”). To create a set of variants for mapping the maternal genome we used the Linux command-line tool “awk” to extract variants with paternal genotype “0/0” and maternal genotype “0/1” (hereafter, the “female PT dataset”). Note that variants with paternal genotype “1/1” were not included because the paternal genome was the reference genome, thus no paternal genotype should be homozygous for the alternative allele. To create a dataset of markers to map the paternal genome we used “awk” to extract variants with paternal genotype “0/1” and maternal genotype “0/0” or “1/1” from the filtered dataset (hereafter, the “male PT dataset”). In this study, we identified 303,020 variants (255,004 SNPs and 48,016 indels) suitable for mapping the maternal genome and 251,912 variants (217,242 SNPs and 34,670 indels) suitable for mapping the paternal genome. SNPs and indels were treated equivalently for mapping purposes.

Next, we used a custom bash script (getPTvariants.sh) to identify probable genotyping errors according to the segregation pattern of offspring genotypes. In both resulting datasets, the segregation of F_1_ genotypes is expected to be 1:1 homozygous:heterozygous. Homozygous offspring should have the same genotype as the homozygous parent (e.g., if one parent is “0/0” and the other parent “0/1”, homozygotes should be “0/0”). An F_1_ offspring with a genotype of the alternative homozygote class (“1/1” in the preceding example) would probably be the result of a genotyping error. We determined the frequencies of such errors for each F_1_ offspring, and found they had an average of 1.18% ± 0.21% (mean ± standard deviation) in the female PT dataset and 1.24% ± 0.19% in the male PT dataset.

Unexpected homozygous genotypes could also arise in F_1_ offspring if one of the two parents were misgenotyped. At such a variant, the unexpected homozygote class should segregate with other genotypes in a Mendelian pattern. To search for this type of genotyping error, we determined the frequency of unexpected homozygous genotypes at each variant in both datasets. We found 5.12% of variants in the female PT dataset and 4.9% of variants in the male PT dataset had unexpected homozygotes. At each of these variants, the number of unexpected homozygotes averaged 24.59% ± 0.68% in the female PT dataset and 23.69% ± 1.13% in the male PT dataset. The occurrence of unexpected homozygotes at a frequency of ~ 0.25 is consistent with both parents being heterozygous at these variants. To exclude these variants, as well as the genotyping errors described above, we changed all unexpected homozygotes to missing data, then excluded variants with more than 10% missing data from each PT dataset. The resulting files, GATKBP-passed.femaleHet.abxabRemoved.vcf and GATKBP-passed.maleHet.abxabRemoved.vcf can be downloaded from https://zenodo.org/record/6368105 [50].

### Genetic map construction

Genetic maps were constructed in R (version 3.6.1) [58] with package OneMap (Version 2.1.1) [59]. Prior to importing the datasets into R/Onemap, variants were tested for Mendelian segregation (χ^2^ goodness of fit) and those where *p* < 0.00001 were removed with a custom Perl script (removeDistorted.pl) The resulting files, femalePT.vcf.gz and malePT.vcf.gz, are available at https://github.com/nicotralab/chen-et-al-sex-determination [49]. Each dataset was then thinned with vcftools [60] to ensure that the distance between adjacent variants was no less than 5000 bp and converted from vcf format into OneMap’s “.raw” format with a bash shell script (thin-for-onemap.v2.sh). After this step, we were left with 23,462 variants (20,058 SNPs and 3404 indels) for the maternal genome and 22,359 variants (19,771 SNPs and 2863 indels) for the paternal genome (Additional Files 10 and 11).

We constructed linkage maps in R (version 3.6.1; [58] with the package OneMap (Version 2.1.1) [59]. Variants with identical genotypes in the F_1_ animals were binned to create single markers for linkage mapping with the function find_bins(). After binning, we recalculated segregation distortion using a Bonferroni-corrected *p*-value of 0.05 and removed any remaining distorted markers. This resulted in a set of 977 markers for the maternal genome and 487 for the paternal genome. Two-point tests were used to calculate recombination fractions and LOD scores for each pair of markers, and linkage groups between non-distorted markers identified with a maximum recombination fraction (rf) of 0.4 and minimum LOD score determined by the OneMap function suggest_lod() (6.14 for the female dataset and 5.56 for the male dataset). In both cases, 15 linkage groups were obtained.

To order markers within each linkage group, the function order_seq() was used. This function selects an initial set of five markers and applies an exhaustive search to determine the order with the lowest LOD score. To this framework map, the remaining markers are added one-by-one to optimize the total LOD score of the growing map. Recombination fractions were converted to gastrozooid (cM) units using the Kosambi map function.

Most of the initial maps contained pairs of markers that were placed within 0.0001 cM of each other by the OneMap software. Upon closer inspection, we discovered that these markers were simply markers that were located on the same contig in the reference genome but had their alternative alleles in opposite phase of one another. Since these markers were essentially redundant to one another we decided to remove them from the maps. To do this we identified them in the initial maps with a custom perl script (identify_redundant_markers.pl), then removed them using the drop_marker function in OneMap.

A recombination fraction plot was then generated with the function rf_graph_table and visually inspected to identify misplaced markers. These were removed from the map and re-inserted with the try_seq() function. Markers that could not be confidently placed were removed entirely from the final maps. Summary statistics for each map were calculated using the Genetic Map Comparator [61]. The “unbinned” maps were created by using the custom perl script unbin_markers_in_map.pl.

### Comparison of recombination rates

To compare recombination rates in the female and male genomes, we identified pairs of markers in the final maps that were located within 5 kb of each other in the reference genome assembly. Linkage groups having two or more such markers were then reconstructed as described for the initial maps. Linkage maps were then compared and summary statistics calculated with the Genetic Map Comparator [61].

### QTL mapping

Loci linked to sexual phenotype were identified using in R with package qtl (version 1.44–9) [28]. Prior to QTL mapping, the data were prepared for R/qtl with a custom perl script (onemapRaw_to_Rqtl.pl). Briefly, for each PT dataset, the marker data in OneMap’s.raw format and the corresponding linkage map were combined and converted to R/qtl’s.csvr format. Header information including the phenotypes (sex) of each colony was added manually. The two resulting files (femaledata.rqtl.with.phenotypes.csvr and maledata.rqtl.with.phenotypes.csvr available in from https://github.com/nicotralab/chen-et-al-sex-determination) were imported into R using the read.cross function. For each dataset, QTL genotype probabilities were calculated with calc.genoprob with default settings and the kosambi map function. A single-QTL genome scan was performed using the function scanone for a binary phenotype under the Haley-Knott regression model, with 1000 permutations and perm.Xsp = 0. Significance thresholds were calculated with the summary function. Figures were created with the plot function, then imported into Adobe Illustrator for further annotation.

### Depth of coverage analysis with SATC

For each sample, raw illumine reads were mapped to the reference genome using BWA-MEM2 [62]**,** and the resulting sam files were sorted and compressed with Samtools [55]. For this mapping, contig HyS0057 was split into three segments to reflect the misassembly from 1,104,702 to 1,185,628 (Additional File 1: Table S1). Duplicates were removed with Picard [56]. Secondary and unmapped reads were removed with Samtools fixmate and the resulting bam file was indexed with Samtools index. Index stats were calculated with Samtools idxstats. SATC was run online via a Shiny app that is available at http://popgen.dk:3838/genis/satc/. The idxstats files for each sample were converted into an input file with the bash script make_shiny_input.sh, which was obtained from http://popgen.dk:3838/genis/satc/. The resulting datafile (Additional file 12) was uploaded and SATC was run with the following options: scaffolds weighted by length, gaussian clustering, and a minimum scaffold length of 2500 bp. Normalization was based on the mean coverage of the five longest contigs in the genome assembly.

### Identification of candidate Y-linked genes

Coordinates from each annotated gene from the reference assembly were used to cut assembled contigs into individual gene contigs. A FASTA file of all gene contigs was then used as a new reference assembly for mapping. Raw Illumina reads from each 339 library were individually mapped to this reference using HISAT2 under default parameters. The resulting sam file was converted to a sorted bam for further analysis. Coverage of each gene was summarized using the samtools coverage function. Coverage statistics were then assessed using a simple python script to identify genes that met our criteria.

### Gene annotation

Gene models were downloaded from the *Hydractinia* genome portal [52]. Gene functions were predicted using the standalone version of PANNZER2 [63] with default parameters, as well as with a DIAMOND [64] similarity search against nr with the option “blastp.” Homologous sequences in the NCBI Model Organisms (landmark) database were identified using BLASTP as implemented through the BLAST website (https://blast.ncbi.nlm.nih.gov/Blast.cgi). Conserved protein domains were identified with the Pfam database using hmmscan as implemented by the HMMER Webserver [65].

### Gene expression analysis

Sex-specific expression of genes in the putative sex determination locus was estimated using previously published RNA-seq libraries from *H. symbiolongicarpus* gastrozooids and gonozooids [32–35]. Here, we reanalyzed the raw sequencing data to identify genes that were expressed only in one type of sexual polyp and in no other polyp type. The paired-end RNA-seq reads from each library were mapped to the entire genome assembly using HISAT2 [66] under default settings. The resulting mapping files were processed and sorted using Samtools [55] before proceeding to quantitation. Using the reference annotations for the primary haplotype of the genome, FPKMs of each gene model were estimated for each library and normalized by library size using the cuffnorm function of Cufflinks [67].

## Supplementary Information

**Additional file 1: Figures S1-S5, Tables S1-S2, S5.** **Fig. S1.** Pedigree. **Fig. S2.** Maternal linkage map. **Fig. S3.** Paternal linkage map. **Fig. S4.** Karyotype. **Fig. S5**. QTL analysis excluding sexual chimeras. **Table S1.** Assembled genome in linkage maps. **Table S2.** Contigs in multiple linkage groups. **Table S5.** Genes with overrepresented coverage in male genomes.

**Additional file 2.** A space-delimited text file representing the maternal linkage map. Columns denote linkage group, marker name, and genetic position in centimorgans.

**Additional file 3.** A space-delimited text file representing the paternal linkage map. Columns denote linkage group, marker name, and genetic position in centimorgans.

**Additional file 4.** A space-delimited text file showing all markers from that can be placed on the maternal linkage map.

**Additional file 5.** A space-delimited text file showing all markers from that can be placed on the paternal linkage map.

**Additional file 6:** **Table S3.** SATC output.

**Additional file 7.** A text file containing FASTA formatted nucleotide sequences of 817 genes located in the non-recombining region of the X and Y chromosomes.

**Additional file 8: Table S4.** A tab-delimited text file with gene expression information and annotations for each candidate sex-determination gene. Each field is described in the comments at the beginning of the file.

**Additional file 9.** A tab delimited text file with per sample sequencing statistics.

**Additional file 10.** A space-delimited text file of genotype data in R/Onemaps “.raw” format representing the female PT dataset.

**Additional file 11.** A space-delimited text file of genotype data in R/Onemaps “.raw” format representing the male PT dataset.

**Additional file 12.** Input file for SATC.

## Data Availability

The dataset(s) supporting the conclusions of this article is(are) available in the BioProject repository, PRJNA807936 (https://www.ncbi.nlm.nih.gov/bioproject/?term=PRJNA807936) and PRJNA816479 (https://www.ncbi.nlm.nih.gov/bioproject/?term=PRJNA816479), Zenodo (https://doi.org/10.5281/zenodo.6584944), and GitHub (https://github.com/nicotralab/chen-et-al-sex-determination, which is archived on Zenodo at https://doi.org/10.5281/zenodo.6369241).

## Abbreviations

DoC: Depth of coverage
FPKM: Fragment per kilobase per million mapped reads
GATK: Genome Analysis Toolkit
SATC: Sex assignment through depth of coverage
SNP: Single nucleotide polymorphism
