# Supplementary material for: XY sex determination in a cnidarian
Source: BMC Biol. 2023 Feb 13;21:32. doi: 10.1186/s12915-023-01532-2 (PMC9926710; doi:10.1186/s12915-023-01532-2)
Supplement: Supplementary file 1 — Additional file 1: Figures S1-S5, Tables S1-S2, S5. Fig. S1. Pedigree. Fig. S2. Maternal linkage map. Fig. S3. Paternal linkage map. Fig. S4. Karyotype. Fig. S5. QTL analysis excluding sexual chimeras. Table S1. Assembled genome in linkage maps. Table S2. Contigs in multiple linkage groups. Table S5. Genes with overrepresented coverage in male genomes. [file 12915_2023_1532_MOESM1_ESM.pdf]

Additional File 1

Supplementary Materials for:

XY sex determination in a cnidarian

Ruoxu Chen[1,2], Steven M. Sanders[3,4], Zhiwei Ma[3,4], Justin Paschall[5], E. Sally Chang[5], Brooke M. Riscoe[3,4], Christine E. Schnitzler[6], Andreas D. Baxevanis[5], and Matthew L. Nicotra[3,4,7,*]

[1]School of Medicine, Tsinghua University, Beijing, China

[2]Visiting Scholar, School of Medicine, University of Pittsburgh, Pittsburgh, PA, USA

[3]Starzl Transplantation Institute, Department of Surgery, University of Pittsburgh, Pittsburgh, PA, USA

[4]Center for Evolutionary Biology and Medicine, University of Pittsburgh, Pittsburgh, PA

[5]Computational and Statistical Genomics Branch, National Human Genome Research Institute, National Institutes of Health, Bethesda, MD, USA

[6]Whitney Laboratory for Marine Bioscience, University of Florida, St. Augustine, FL, USA

[7]Department of Immunology, University of Pittsburgh, Pittsburgh, PA, USA

*Corresponding Author: Matthew L. Nicotra

E-mail: matthew.nicotra@pitt.edu

This PDF file includes:

Figures S1 to S5
Tables S1, S2, and S5
Descriptions of Additional Files 2-12

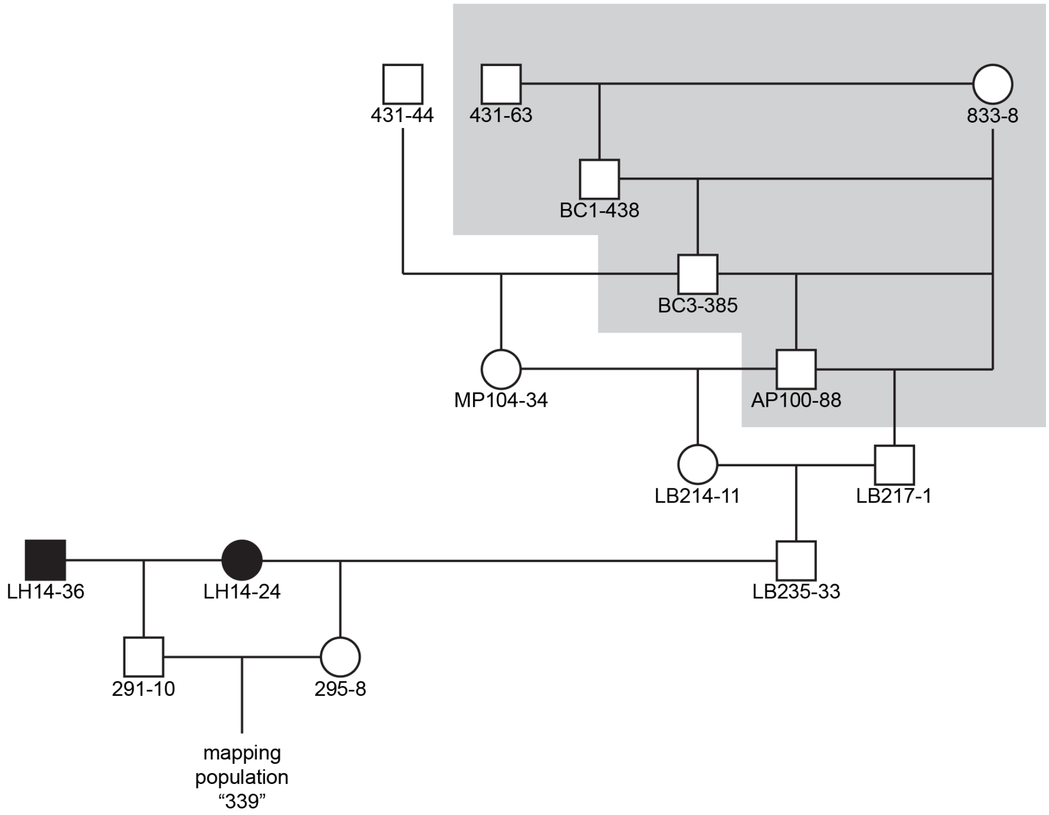

Fig S1. Pedigree of the colonies used to generate the mapping population.
Field-collected colonies are denoted with black symbols. Colony 291-10 is the offspring of two colonies collected from Lighthouse Point, New Haven, CT in 2014. Colony 295-8 is the offspring of a field collected colony and a laboratory strain, 235-33. The pedigree of colony 235-33 can be recreated by concatenating previously published pedigrees (shaded area) (Cadavid et al. 2004; Powell et al. 2007). Colony AP100-88 is from the mapping population in Powell et al. (2007). Colony 431-44 is from the mapping population in Cadavid et al. (2004).

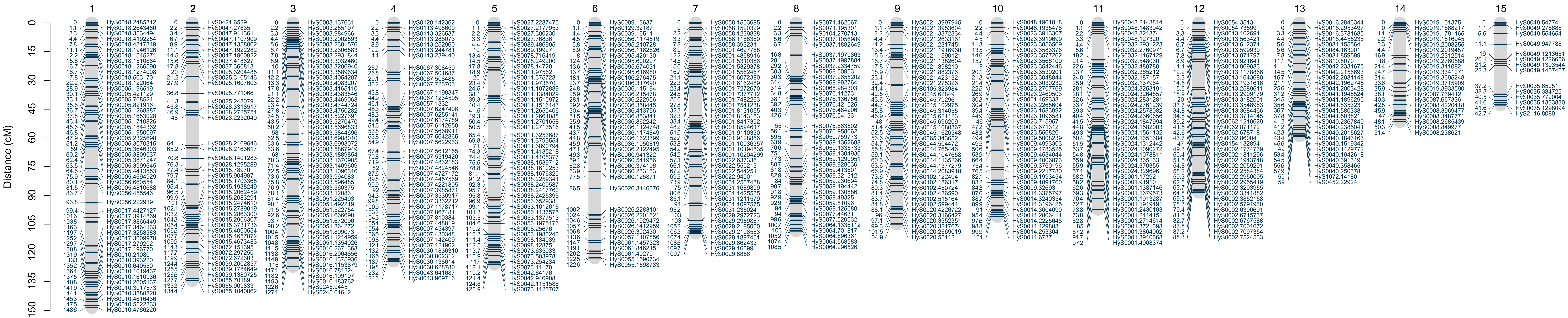

Fig. S2. Maternal linkage map of the Hydractinia genome. Marker names (contigname.position) are displayed to the right of each linkage group. Genetic position, in centimorgans, is displayed to the left.

(Previous Page)

Fig. S2. Maternal linkage map of the *Hydractinia* genome. Marker names (contigname.position) are displayed to the right of each linkage group. Genetic position, in centimorgans, is displayed to the left.

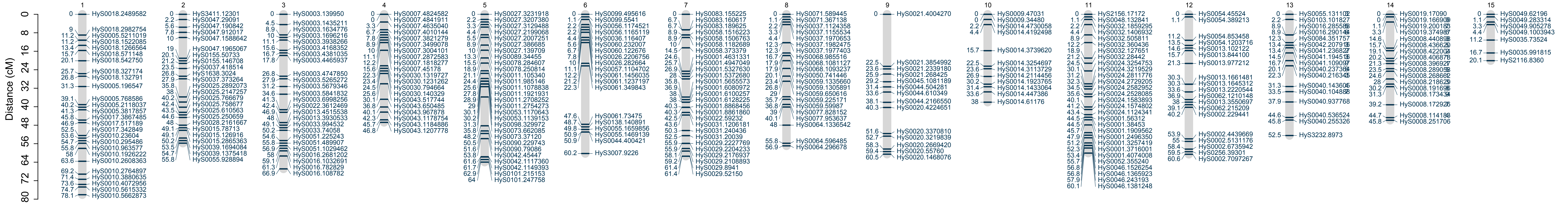

Fig. S3. Paternal linkage map of the Hydractinia genome. Marker names (contigname.position) are displayed to the right of each linkage group. Genetic position, in centimorgans, is displayed to the left.

(Previous Page)

Fig. S3. Paternal linkage map of the *Hydractinia* genome. Marker names (contigname.position) are displayed to the right of each linkage group. Genetic position, in centimorgans, is displayed to the left.

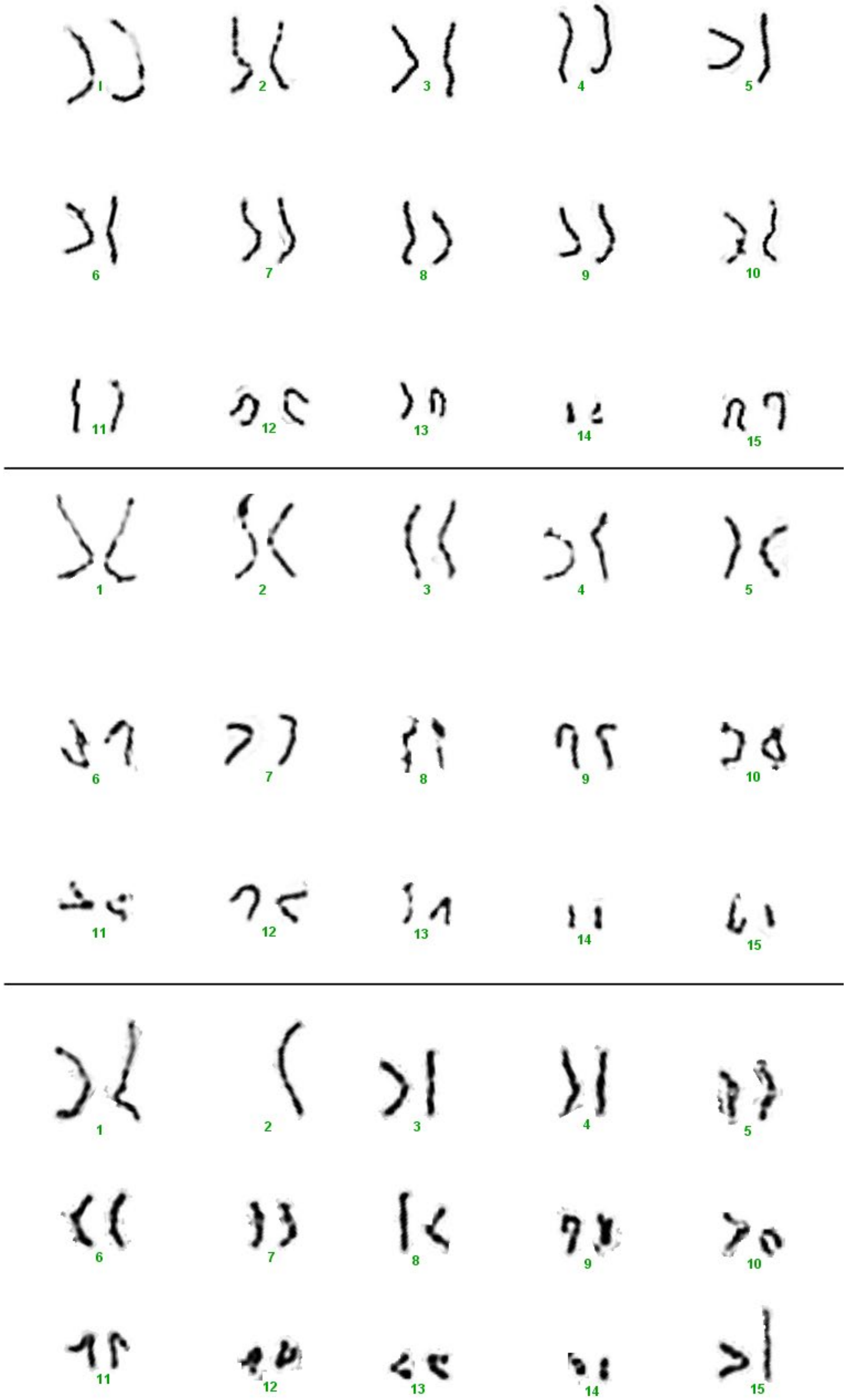

Fig S4. Karyotype analysis of *Hydractinia symbiolongicarpus*
Karyotype of three metaphase cells from 64-128 cell embryos. Despite poor G-banding, each had what appeared to be a normal diploid chromosome competent of 15 pairs (2n = 30). Note that the numbers assigned to each pair of chromatids *do not* correspond to the numbers assigned to linkage groups.

A

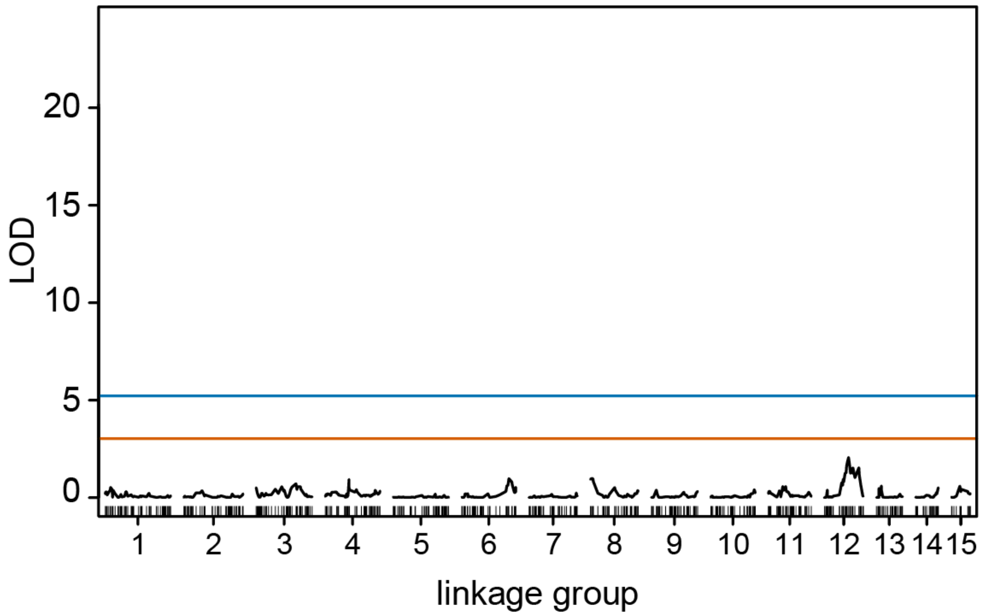

B

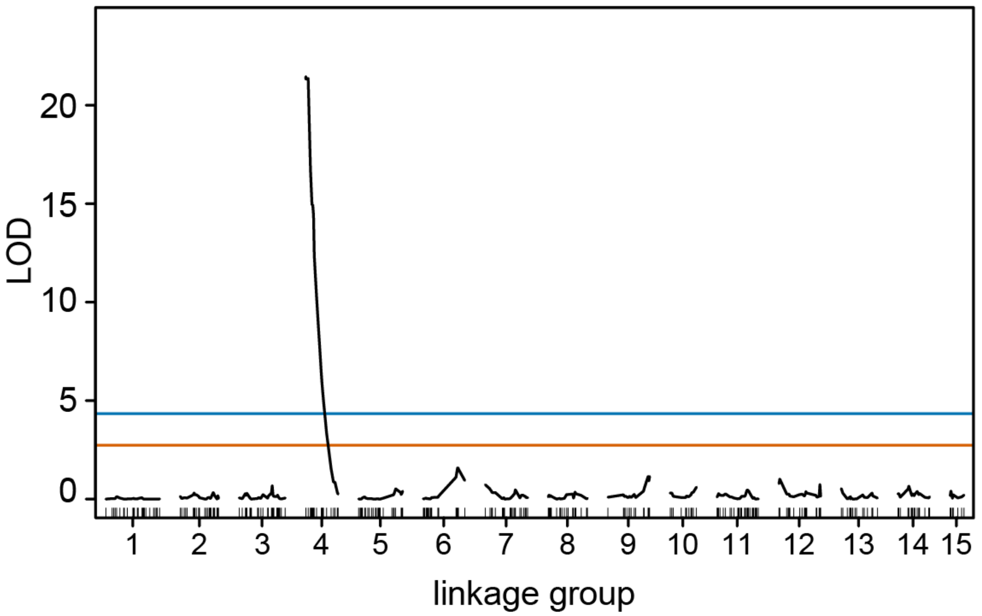

Fig. S5. QTL analysis excluding sexual chimeras.
(A) LOD chart of QTL for sex in the maternal linkage map. *(B)* LOD chart of QTL for sex in the paternal linkage map. The blue

Table S1. Representation of the assembled *Hydractinia* genome in linkage maps

placement in linkage maps	contigs		base pairs		annotated genes	
both	160	*(3.3)**	265,235,647	*(65.2)*	17,444	*(79.2)*
maternal only	72	*(1.5)*	3,850,356	*(0.9)*	127	*(0.6)*
paternal only	41	*(0.8)*	3,770,032	*(0.9)*	186	*(0.8)*
none	4,567	*(94.4)*	133,837,400	*(32.9)*	4,265	*(19.4)*
**TOTAL**	**4,840**		**406,693,435**		**22,022**	

* percent of total indicated in parentheses

Table S2. Contigs with variants in multiple linkage groups

**Contig**	**Linkage group in maternal map**	**Linkage group in paternal map**
HyS0001	7, 11	7, 11
HyS0013	3, 12	3, 12
HyS0016	3, 13	3, 13
HyS0022	3, 7	3, 7
HyS0030	4, 11	4, 11
HyS0037	2, 8	2, 8
HyS0039	2, 6, 10	2, 10
HyS0042	3, 5, 13	5, 13
HyS0043	2, 4	2, 4
HyS0048	10, 11	10, 11
HyS0050	8, 10	8, 10
HyS0055	2, 6	2, 6
HyS0056	1, 6	1, 6
HyS0057	4, 6	4, 6
HyS0122	10, 14	10, 14

Table S3: Contigs identified by SATC (Additional File 6)

Table S4: Expression and homology of candidate sex determination genes (Additional File 8)

Table S5: Genes with a <50% coverage in females and >50% coverage in males.

	% of gene covered in female samples (n = 40)		% of gene covered in male samples (n = 47)	
**Gene ID**	**Mean**	**Standard deviation**	**Mean**	**Standard deviation**
HyS0007.272	24.4	25.1	95.9	16.4
HyS0007.274	27.3	23.5	95.7	17.1
HyS0057.88	17.9	5.1	98.6	0.8
HyS0057.89	23.1	10.9	99.5	0.5
HyS0057.124	36.3	7.7	99.9	0.4
HyS0067.1	18.5	13.8	99.7	0.9
HyS0067.6	23.8	20.1	99.8	0.5
HyS0070.87	0.0	0.0	99.6	0.8
HyS0070.92	4.8	18.2	98.8	2.7
HyS0070.105	19.2	5.5	99.9	0.2
HyS0070.115	0.0	0.0	100.0	0.1
HyS0070.117	49.4	10.0	99.9	0.5
HyS0113.2	16.2	2.7	100.0	0.1
HyS0113.14	21.3	1.2	99.5	0.2
HyS0113.15	3.6	1.4	100.0	0.0
HyS0113.16	4.9	2.7	98.2	0.2
HyS0113.42	36.8	26.0	100.0	0.1
HyS3947.3	27.9	9.0	99.0	0.5

Table S6. Summary of expression of candidate genes

Polyp type	Number of genes
Gastrozooids and gonozooids (male, female, or both)	315
Gastrozooids only	101
Female and male gonozooids	4
Female gonozooids only	9
Male gonozooids only	29
None	359
**TOTAL**	**817**
